# Transcutaneous measurement of renal function in two rodent models of obstructive nephropathy

**DOI:** 10.1186/s13104-023-06387-y

**Published:** 2023-06-26

**Authors:** Michael Schou Jensen, Isabela Bastos Binotti Abreu de Araujo, Henricus A.M. Mutsaers, Rikke Nørregaard

**Affiliations:** Department of Clinical Medicine, Aarhus University, Palle Juul-Jensens Boulevard 11, Aarhus N, DK-8200 Denmark

**Keywords:** Chronic kidney disease, Acute kidney injury, Glomerular filtration rate, Renal function, Plasma creatinine, Obstructive nephropathy, The 3Rs.

## Abstract

**Objective:**

Glomerular filtration rate (GFR) is a key indicator of renal function. In both clinical practice and pre-clinical research, serum levels of endogenous filtration markers, such as creatinine, are often used to estimate GFR. However, these markers often do not reflect minor changes in renal function. In this study, we therefore set out to evaluate the applicability of transcutaneous GFR (tGFR) measurements to monitor the changes in renal function, as compared to plasma creatinine (pCreatinine), in two models of obstructive nephropathy, namely unilateral ureteral obstruction (UUO) or bilateral ureteral obstruction followed by release (BUO-R) in male Wistar rats.

**Results:**

UUO animals showed a significant reduction in tGFR compared to baseline; whereas pCreatinine levels were not significantly changed. In BUO animals, tGFR drops 24 h post BUO and remains lower upon release of the obstruction until day 11. Concomitantly, pCreatinine levels were also increased 24 h after obstruction and 24 h post release, however after 4 days, pCreatinine returned to baseline levels. In conclusion, this study revealed that the tGFR method is superior at detecting minor changes in renal function as compared to pCreatinine measurements.

**Supplementary Information:**

The online version contains supplementary material available at 10.1186/s13104-023-06387-y.

## Introduction

Glomerular filtration rate (GFR) is a key indicator of renal function and widely used for the diagnosis and staging of kidney disease. In clinical practice, serum levels of endogenous filtration markers, such as creatinine or cystatin C, are often used to approximate renal function, and to improve accuracy, equations for estimated GFR (eGFR) incorporate demographic variables such as age, weight and sex [1–3]. However, eGFR does not always adequately reflect renal function, especially when the GFR is close to the normal range, making early detection of kidney injury very complicated [4]. Pre-clinical studies face similar issues, since commonly used serum markers in animals also fail to capture minor changes in renal function. In general, approximately 50% of renal function must be lost before serum creatinine levels increase [5, 6]. Moreover, increased serum creatinine does not necessarily reflect renal damage, but may be caused by hypovolemia, dehydration, protein catabolism or a combination thereof. These extra-renal elements should be considered when using creatinine as an experimental endpoint for renal pathology [6]. To accurately monitor GFR in laboratory animals the employed method should reflect GFR in real-time, detect minor changes, and not be stressful. Traditional methods to measure GFR are often based on ^51^Cr-EDTA, inulin, iodixanol, blood urea nitrogen or creatinine clearance, all of which are invasive, imprecise and labor intensive [7–11]. Additionally, the majority of these techniques require deep anesthesia, which can influence renal hemodynamics and thus renal function [12, 13]. Moreover, repeated blood and urine sampling greatly impacts animal welfare. To simplify and improve GFR assessment, a new technique was developed that allows for the transcutaneous measurement of the elimination kinetics of fluorescein isothiocyanate (FITC)-sinistrin in freely moving mice and rats [10, 14, 15].

Within the field of nephrological research, ureteral obstruction – either unilateral ureteral obstruction (UUO) or bilateral ureteral obstruction (BUO) – is one of the most frequently used models for kidney disease. UUO [16, 17] is generally used as a chronic kidney disease (CKD) model, whereas BUO is mainly used to model acute kidney injury (AKI). Both models are based on surgical obstruction of the ureter, which can be experimentally manipulated with respect to timing, severity, and duration, while reversal of the obstruction permits the study of recovery. Ureteral obstruction markedly impairs renal function, including GFR [17–20].

In this study, we therefore set out to evaluate the applicability of transcutaneous GFR (tGFR) measurements to monitor the changes in renal function, as compared to plasma creatinine (pCreatinine), in two models of obstructive nephropathy, namely UUO and BUO. We hypothesize that the tGFR method is more accurate and more sensitive to minor changes in GFR than pCreatinine.

## Main text

## Materials and methods

### Experimental design and surgical procedures

Experiments were performed using a total of twenty-eight 7 to 8-week-old male rats, *Rattus norvegicus, Wistar* (Janvier Labs, Le Genest-Saint-Isle, France). Animals had free access to water and standard rodent chow (Altromin, Lage, Germany). Rats were housed in pairs in a controlled environment; 12 h:12 h light-dark cycle, constant temperature of 21 ± 2 °C, and humidity of 55 ± 2%. Standard housing conditions and husbandry procedures were identical across the control and experimental groups. The rats were cared for daily and monitored for pain and distress between and after the procedures using a general distress scoring sheet. The rats were allowed to acclimate at least one week prior to the surgical procedures.

Animals were randomly assigned to the experimental groups, and sample size calculation was based on a pilot study. During surgery, the animals were anesthetized with 2% sevoflurane (Sevorane, AbbVie, Copenhagen, Denmark) mixed with atmospheric air at 2 L/min, and injected s.c. with 50 µg/kg buprenorphine (Temgesic, Indivior UK Limited, Berkshire, UK) in order to minimize postoperative pain. In addition, buprenorphine (7.5 µg/ml) was added to the drinking water to maintain analgesia for 48 h post-surgery.

UUO surgery was performed as previously described [21], and the obstruction was maintained for 11 days (11dUUO; n = 8): which is sufficient to induce severe renal impairment. Sham-operated rats (n = 7) were included as control. One UUO animal died due to an unknown cause in the days after the surgery. BUO surgery was performed as described previously [20, 22], with slight modifications. Briefly, rats were placed on a heating pad and laparotomy was performed to expose both ureters. Subsequently, a latex elastomer (AgnTho’s, Lidingö, Sweden) was placed around the midportion of each ureter, and the ureter was then occluded by tightening the elastomer with a 4-0 silk non-absorbable suture (Softsilk, Medtronic, Watford, UK). After which, rats were housed individually in metabolic cages for 24 h (24hO). Subsequently, both ureters were released (R), and the rats were placed in the metabolic cages for another 24 h to measure urine output. BUO-R animals were monitored for an additional 11 days (11dR; n = 7): which is sufficient time to observe recovery. Sham-operated animals (n = 6) were included as control. One Sham animal was excluded from all analyses due to lasting postoperative discomfort.

### Transcutaneous glomerular filtration rate (tGFR)

The kidney function was assessed by tGFR measurements as previously described [10]. Shortly, under sevoflurane anesthesia, part of the back of the rats was depilated and a non-invasive clearance (NIC)-Kidney device (MediBeacon, Mannheim, Germany) was attached to the area using an adhesive patch. The measurement started with a background reading of 1 to 4 min before 0.35 mg/g body weight (BW) fluoresceine isothiocynate sinistrin (FTIC-S; Fresenius Kabi, Graz, Austria) was intravenously administrated. During 2 h of measurement, each animal was housed individually, free to move, and had access to water and food. GFR (ml/min/100 g BW) was calculated from the transcutaneous measurement of FITC-S using a 3-compartment model [23]. In the UUO experiment, tGFR measurements were performed on the following timepoints: before surgery (baseline); 24 h after obstruction; and on day 4, 7 and 11 after obstruction. In the BUO-R experiment, tGFR measurements were performed on the following timepoints: before surgery (baseline); 24 h after obstruction; 24 h after release; and on day 4, 7 and 11 after release of the obstruction. No measures of blinding or randomization were taken.

### Plasma and urine creatinine test

Blood (50 µl) was collected from the tail vein immediately prior to the tGFR measurements, when the rats were under anesthesia. After the final tGFR measurement, blood was collected via cardiac puncture performed as a terminal procedure under sevoflurane anesthesia followed by cervical dislocation. Plasma creatinine levels were determined using Creatinine Assay Kit (Sigma-Aldrich, Schnelldorf, Germany), according to the manufacturer’s instructions. Urine creatinine was measured using a Roche Cobas 6000 analyzer (Roche Diagnostics, Risch-Rotkreuz, Switzerland). Creatinine clearance was calculated using the following formula:

$$\frac{Urine Creatinine * Urine Volume}{Plasma Creatinine}$$

Data was expressed pr. 100g BW [11, 24].

### Statistical analysis

Results are expressed as mean ± standard error of the mean (SEM). Statistics were performed with Graphpad Prism 9.4.1 (Graphpad Software, Inc., San Diego, CA, USA). A repeated measure one-way ANOVA with Dunnett’s multiple comparisons test was performed to compare intragroup kidney function at each time point. Similar analyses were performed for plasma creatinine data. A two-tailed Pearson correlation model was used to compare the relationship between tGFR and pCreatinine. Descriptive data in supplemental tables are presented as mean ± standard error of deviation (SD). Differences between groups were considered to be statistically significant when p < 0.05.

## Results

### Evaluation of renal function in UUO rats

**Fig. 1:**
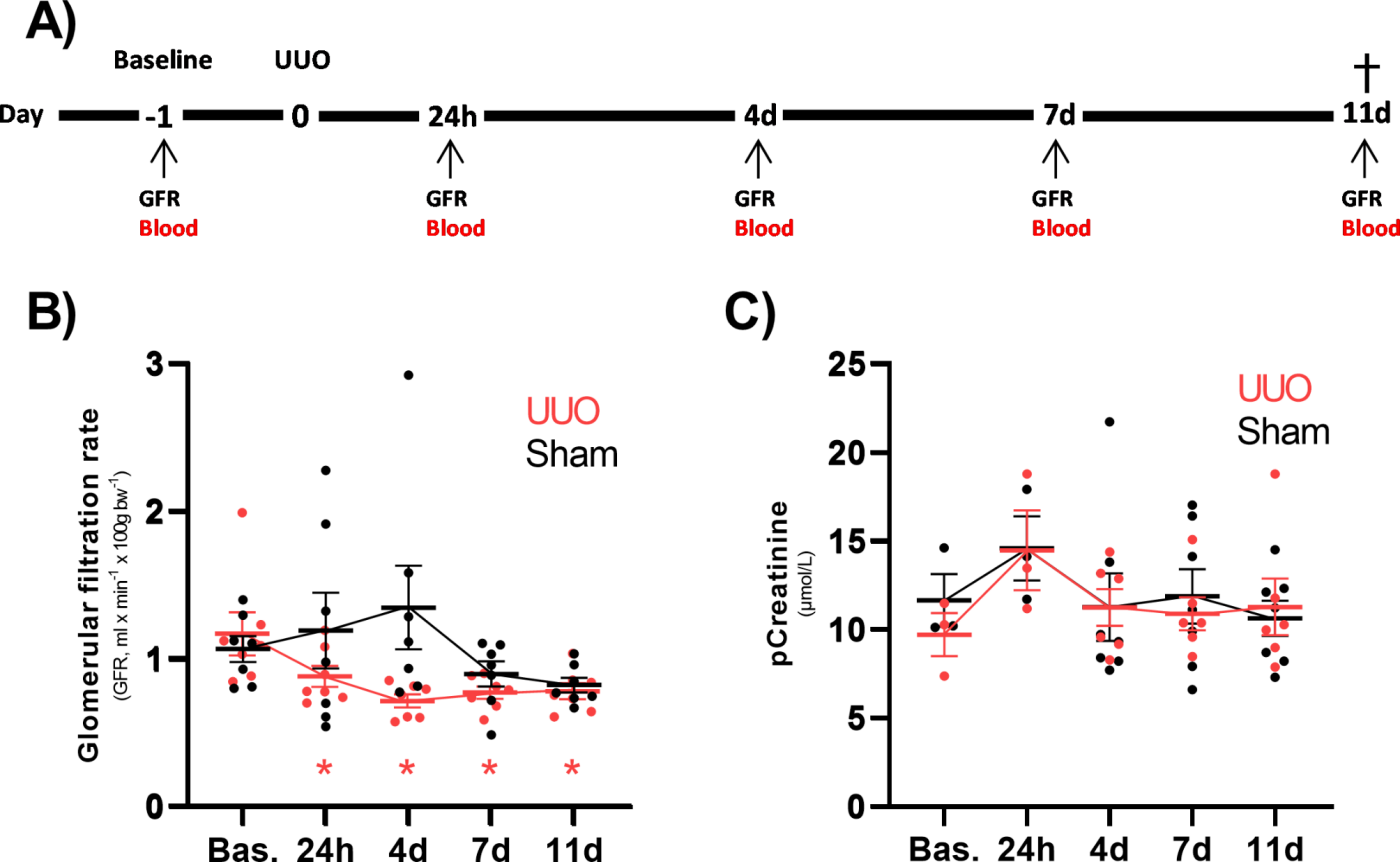
The impact of unilateral ureteral obstruction on tGFR and pCreatinine. Rats were subjected to 11 days of UUO, or Sham surgery, and kidney function was monitored using plasma Creatinine (µmol/L) and transcutaneous GFR measurements (ml/min/100g BW). **(A)** Timeline of experiment. **(B)** tGFR in UUO (n = 7) and Sham (n = 7) rats. **(C)** pCreatinine levels in UUO (n = 3–6) and Sham (n = 3–7) rats. All time points are compared to the corresponding intragroup baseline, data are presented as mean ± SEM. *P < 0.05 compared to UUO baseline

To evaluate the impact of UUO on GFR we utilized the UUO model combined with sequential tGFR measurements and blood sampling, see Fig. 1A for experiment design. Following surgery, we did not observe any significant changes in bodyweight when comparing UUO and Sham groups (Supplemental Table 1). As shown in Fig. 1B, GFR is significantly reduced in UUO animals at all time points after obstruction compared to baseline, whereas no significant changes are observed in Sham animals (Fig. 1B). Moreover, we did not observe any significant differences in pCreatinine following surgery in both UUO and Sham animals as compared to baseline (Fig. 1C). These results indicate a lower sensitivity of pCreatinine to evaluate renal function in the UUO model as compared to tGFR.

**Fig. 2:**
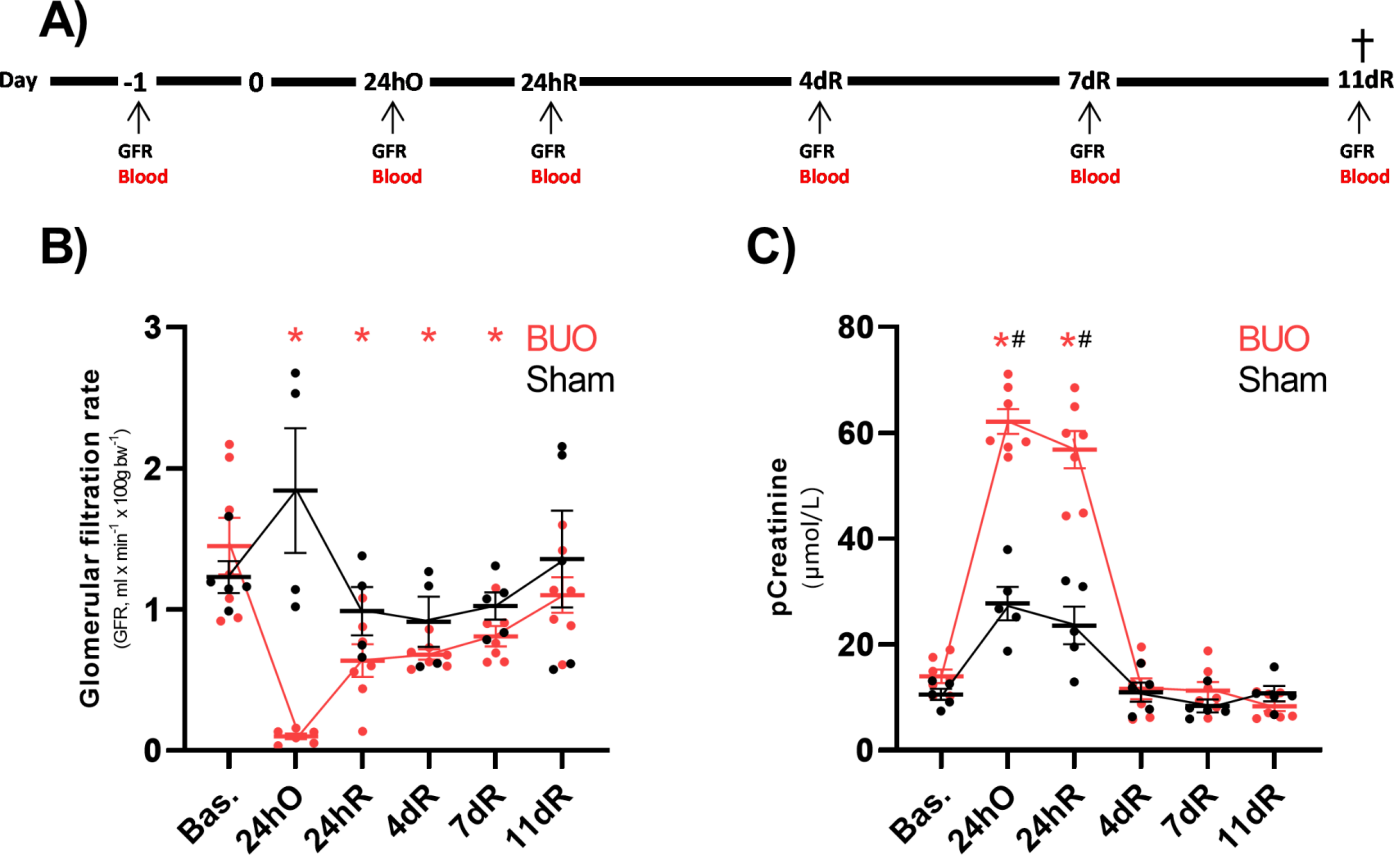
The impact of bilateral ureteral obstruction on tGFR and pCreatinine. Rats were subjected to 24 h of BUO (24hO) followed by release (24 h) of the obstruction, or Sham surgery, and monitored for an additional 11 days. Changes in kidney function were monitored using plasma Creatinine (µmol/L) and transcutaneous GFR (ml/min/100g BW) measurements. **(A)** Timeline of experiment. **(B)** tGFR in BUO-R (n = 6–7) and Sham rats (n = 4–5). **(C)** pCreatinine levels in BUO-R (n = 7) and Sham rats (n = 5). All time points are compared to the corresponding intragroup baseline, data are presented as mean ± SEM. *P < 0.05 compared to BUO baseline. #P < 0.05 compared to Sham baseline

### Evaluation of renal function in BUO-R rats

Next, we evaluated the impact of BUO and subsequent release on GFR; see Fig. 2A for experiment design. Following surgery, we did not observe any significant changes in bodyweight when comparing BUO-R and Sham groups (Supplemental Table 1). Of note, urine excretion was markedly elevated at 24 h post release, indicating successful release of the obstruction, at this time point we also observed a marked reduction in GFR based on creatinine clearance (Supplemental Table 1). As expected, Fig. 2B shows that the GFR drops dramatically 24 h post BUO, whereas Sham animals are not affected in a similar manner (Fig. 2B). The GFR appears to be consistently lower in the BUO group after release of obstruction, when comparing to baseline levels, yet after 11 days the GFR appears to approach baseline levels and is not significant different. Levels of pCreatinine are markedly increased after 24 h of obstruction and 24 h post release; however, after 4 days, pCreatinine levels seem to return to baseline (Fig. 2C). A similar, but less pronounced effect is observed in the Sham animals (Fig. 2C).

### Correlation between tGFR and pCreatinine

**Fig. 3:**
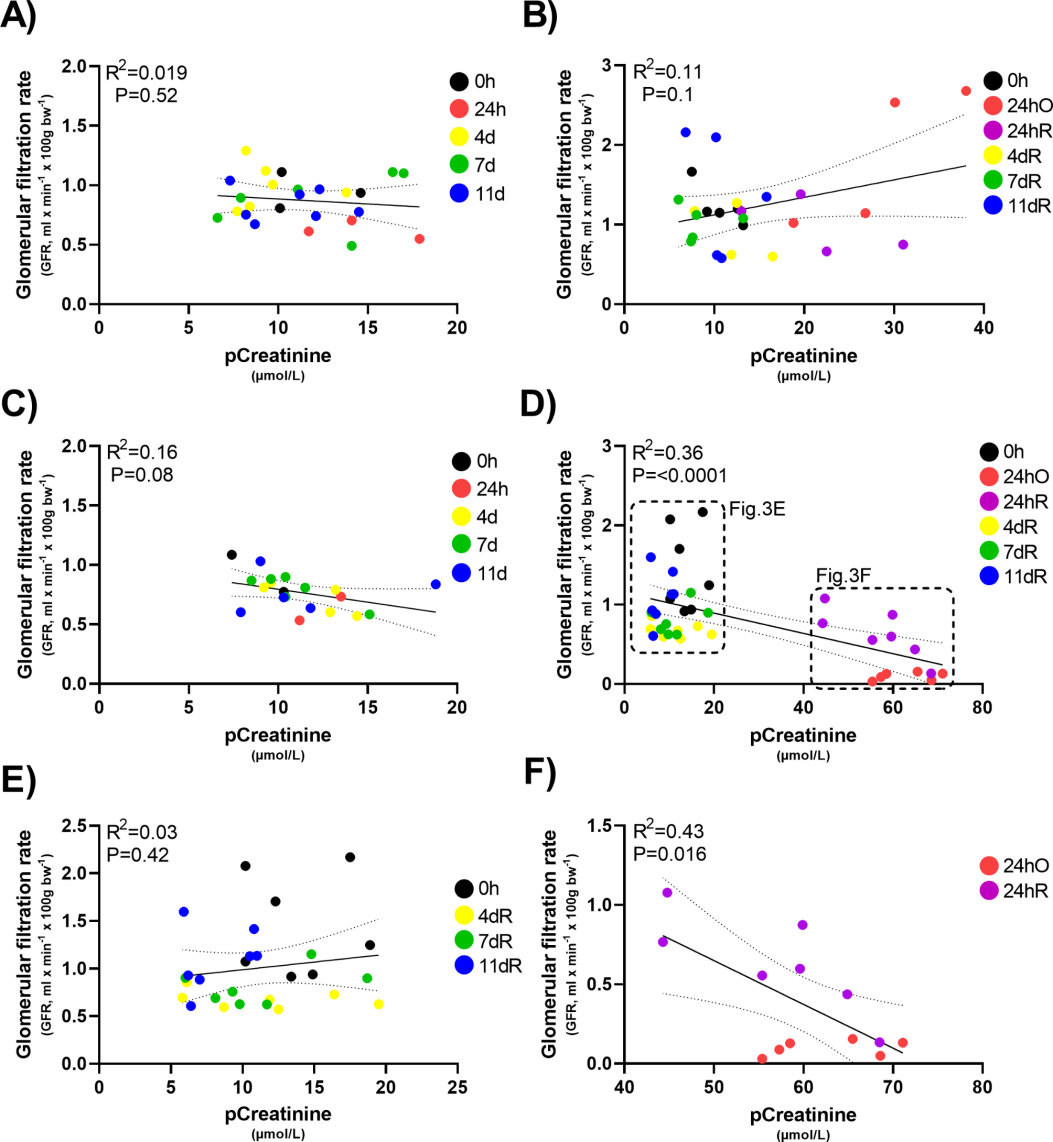
Relationship between tGFR and pCreatinine. Pearson correlation analysis of the relationship between tGFR (ml/min/100g BW) and pCreatinine (µmol/L) in **(A and B)** Sham rats (n = 3–7), **(C)** UUO rats (n = 2–6), and **(D)** BUO-R rats (n = 6–7). Subanalysis of the correlation between tGFR and pCreatinine in BUO-R rats with pCreatinine **(E)** < 20 µmol/L (n = 7) or **(F)** > 40 µmol/L (n = 6–7)

To evaluate if there is a relationship between tGFR and pCreatinine, we performed a Pearson correlation analysis. As shown in Fig. 3A and B, there is no correlation between tGFR and pCreatinine in either UUO Sham (R^2^ = 0.019, P = 0.52) or BUO Sham animals (R^2^ = 0.11, P = 0.1) at any time point. Similarly, there is no noticeable relationship between tGFR and pCreatinine in UUO animals (R^2^ = 0.16, P = 0.08; Fig. 3C). Conversely, a moderate negative correlation was observed between tGFR and pCreatinine in the BUO model (R^2^ = 0.36, P = < 0.0001; Fig. 3D); however, the analysis appears to be highly influenced by the 24hO (red) and 24hR (purple) time points. These data suggest that tGFR and pCreatinine only correlate when renal function in severely impacted. Indeed, no correlation was observed between tGFR and pCreatinine when comparing 0h, 4dR, 7dR and 11dR (Fig. 3E); whereas, a significant correlation (R^2^ = 0.43, P = 0.016) was observed when comparing tGFR and pCreatinine at 24hO and 24hR (Fig. 3F).

## Discussion and conclusion

In the present study, we monitored changes in renal function using pCreatinine measurements and transcutaneous assessment of clearance following both UUO and BUO-R. About a decade ago, the first papers were published showcasing the feasibility of measuring GFR through the skin in conscious rats and mice using FITC-sinistrin and a miniaturized optical device [10, 15]. Since then, the method has also been applied in numerous rodent models of renal pathology, including: unilateral nephrectomy, adenine-induced chronic kidney disease, (poly)cystic kidney disease, hypertensive renal injury, diabetic nephropathy, recurrent dehydration-induced kidney disease, ischemic acute kidney injury, sepsis-induced kidney injury, aging and K^+^ overload [10, 15, 25–39]. However, to the best of our knowledge, our current observations are the first available measurements of GFR, using the NIC-Kidney device, across an 11-day experimental ureteral obstruction protocol in rats. During this timeframe, we observed that the method was well tolerated by all animals. After recovering from sevoflurane anesthesia, the NIC-Kidney device had no impact on movement or behavior of the animals. This indicates a low level of stress in the rats in contrasts to the stress induced by classical clearance studies, which require repeated restrainment and blood sampling [40]. Another advantage of the tGFR method is that it does not rely on accurately timed urine sampling making it a more efficient and faster technique compared with creatinine clearance studies, which often require 24-hour urine collection.

Our findings indicate that UUO and BUO have a substantial impact on GFR, which is not consistently reflected by pCreatinine. Indeed, Pearson correlation analysis revealed that there is no, or only a moderate, correlation between pCreatinine and GFR, indicating that pCreatinine is less sensitive to changes in renal function as compared to tGFR measurements. Our results corroborate previous studies, which used various animal models of AKI and CKD, all showing that the tGFR method is superior for monitoring renal function as compared to pCreatinine or albumin measurements [28, 41–44]. Of note, a spike in pCreatinine was observed in both UUO and BUO-R rats 24 h after surgery, which might be due to post-surgical stress [45].

In the UUO model, we did not see any significant changes in pCreatinine, not even at day 11, when the UUO kidney was clearly fibrotic (Supplemental Fig. 1). This discrepancy might be due to contralateral compensation of the non-obstructed kidney as observed in previous publications [46–49]. Due to this physiological response, it is also not feasible to adequately assess changes in renal function by measuring creatinine clearance in UUO animals. This supports our notion that the tGFR methodology is superior to creatinine clearance.

It is widely acknowledged that GFR is a key indicator of renal health in both clinical practice and pre-clinical research. Yet, even in medicine, there is still a debate regarding the best ways to calculate GFR [50], and there is even less consensus in pre-clinical research. Based on previous work [1, 23, 41, 51, 52], and our own observations, we believe that tGFR measurements currently provide the highest level of evidence concerning renal function and should therefore be incorporated in animal studies related to renal impairment.

In summary, the results obtained in this study support the notion that the tGFR method is a reproducible and appropriate tool for monitoring renal function in rodent models of obstructive nephropathy. Thus, we recommend the method to be employed in future studies in which conscious and freely moving rodents are needed for determining GFR.

### Limitations

In our UUO study, we were only able to determine pCreatinine in three animals at baseline and 24 h after surgery due to hemolysis of the other blood samples. However, we believe that this only slightly impacted the statistical power of our analysis.

When performing tGFR measurements, one must consider day-to-day variability in GFR. This is mostly pronounced in healthy animals and is caused by changes in food intake, hydration status, blood pressure or renal flow [6, 53].

In the current study, we did not conduct a comparative analysis between tGFR and GFR estimated via creatinine clearance. Future studies should include such an analysis in order to fully support our observation that the tGFR method is more sensitive to minute changes in renal function compared with traditional methods to measure GFR.

## Electronic supplementary material

Below is the link to the electronic supplementary material.

Supplementary Material 1: Table S1, Table S2 and Figure S1

## Data Availability

The datasets used and/or analyzed during the current study are available from the corresponding author on reasonable request.

## List of abbreviations

^51^C-EDTA: Chromium-51 labeled ethylenediamine tetraacetic acid
AKI: Acute kidney injury
BUO: Bilateral ureteral obstruction
CKD: Chronic kidney disease
eGFR: Estimated glomerular filtration rate
FITC-S: Fluorescein isothiocyanate-sinistrin
R: Release
tGFR: Transdermal glomerular filtration rate
UUO: Unilateral ureteral obstruction
