# Supplementary material for: Transcutaneous measurement of renal function in two rodent models of obstructive nephropathy
Source: BMC Res Notes. 2023 Jun 26;16:119. doi: 10.1186/s13104-023-06387-y (PMC10294388; doi:10.1186/s13104-023-06387-y)
Supplement: Supplementary file 1 — Supplementary Material 1: Table S1, Table S2 and Figure S1 [file 13104_2023_6387_MOESM1_ESM.pdf]

**Table 1** - Bodyweight		
Timepoint	Sham	UUO
Baseline	296.7 ± 25.7	279.6 ± 11.1
Surgery	301.4 ± 23.9	285.1 ± 7.7
24h	302.1 ± 24.5	285.9 ± 7.4
4d	300.4 ± 23.2	282.6 ± 9.9
7d	313.3 ± 23.2	289.4 ± 8.5
11d	342. 7 ± 18.5	308.6 ± 10.2

Supplemental Table 1 Bodyweight of UUO rats

Body weight (g, mean ± SD) of Sham (n=7) and UUO (n=7) rats.

**Table 2** - Bodyweight (g), urine output (mL) and creatinine clearance (ml x min^{-1} x 100g bw^{-1})		
Timepoints	Sham	BUO
Baseline	285.8 ± 70.6	232.3 ± 44.4
Surgery	281.4 ± 65.4	233 ± 42.2
24hO	270.2 ± 64.2	233.7 ± 43.5
24hR	268.2 ± 57.8	206.9 ± 47.2
4dR	276.2 ± 62.4	216 ± 42.7
7dR	289.8 ± 62.5	243.7 ± 35.5
11dR	318.6 ± 58.2	270.6 ± 30.7
Urine output (mL)	Sham	BUO
24hR	9.83 ± 3.19	40.86 ± 11.26*
Creatinine clearance	Sham (n=5)	BUO (n=5)
24hR	0.54 ± 0.72	0.32 ± 0.64*

Supplemental Table 2 Bodyweight and urine output of BUO-R rats

Body weight (g, mean ± SD) of Sham (n=5) and BUO-R (n=7) rats. Urine output of Sham (n=6) and BUO-R (n=7) measured for 24h after release of obstruction (mL, mean ± SD). Creatinine clearance (ml x min^{-1} x 100g bw^{-1}), mean ± SD) of Sham (n=5) and BUO-R (n=5). Students t test compared to Sham *P < 0.05.

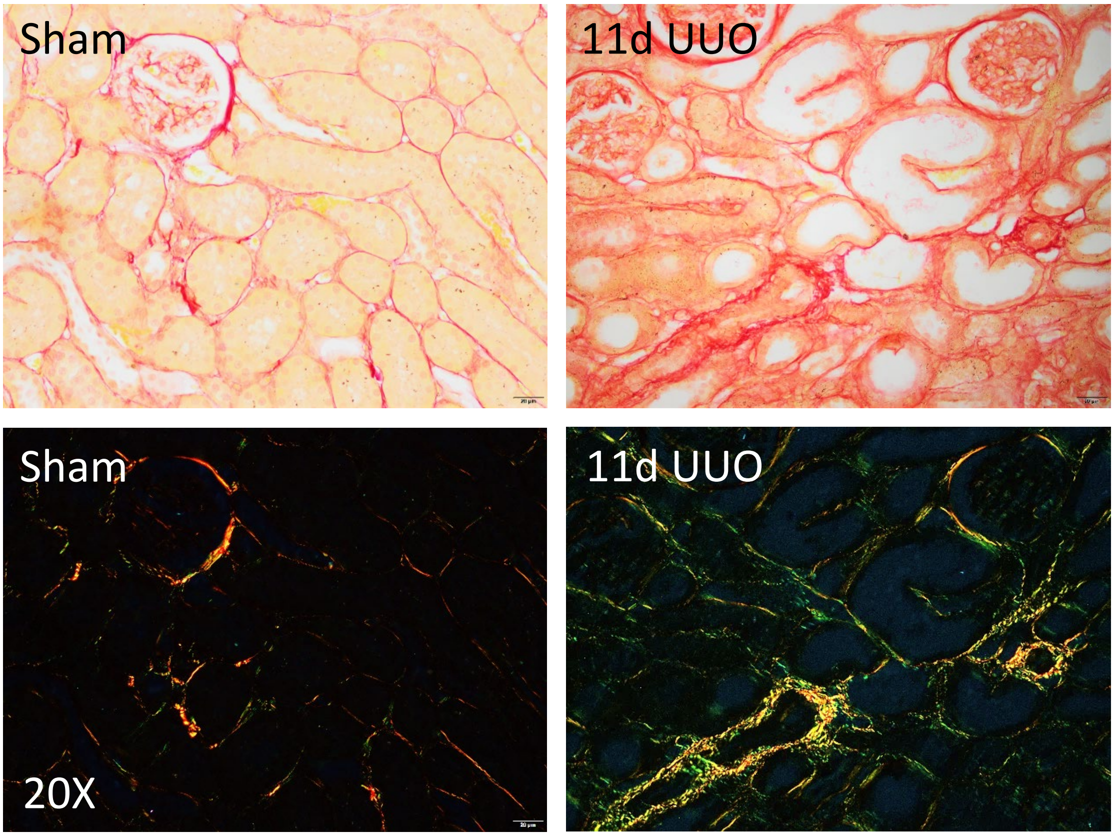

Supplemental Figure 1 Representative Picro-Sirius Red images showing deposition of collagen

UUO kidneys were collected at day 11, cut along the axial plane, and processed for histology. Paraffin-embedded sections (3 µm) were stained with Picro-Sirius Red (Ampliqon, Odense, Denmark). Pictures were taken at 20x magnification using an Olympus BX50 microscope (Olympus Optical Co. Ltd., Nagano, Japan) with and without polarized filter (Olympus Optical Co. Ltd.).
